# Application of laser microdissection to identify the mycorrhizal fungi that establish arbuscules inside root cells

**DOI:** 10.3389/fpls.2013.00135

**Published:** 2013-05-09

**Authors:** Andrea Berruti, Roberto Borriello, Erica Lumini, Valentina Scariot, Valeria Bianciotto, Raffaella Balestrini

**Affiliations:** ^1^National Research Council, Plant Protection Institute - Turin UOSTorino, Italy; ^2^Department of Agricultural, Forest and Food Sciences, University of TorinoTorino, Italy

**Keywords:** AM symbiosis, arbuscules, Glomeromycota, AMF community, ribosomal gene

## Abstract

Obligate symbiotic fungi that form arbuscular mycorrhizae (AMF; belonging to the Glomeromycota phylum) are some of the most important soil microorganisms. AMFs facilitate mineral nutrient uptake from the soil, in exchange for plant-assimilated carbon, and promote water-stress tolerance and resistance to certain diseases. AMFs colonize the root by producing inter- and intra-cellular hyphae. When the fungus penetrates the inner cortical cells, it produces a complex ramified structure called arbuscule, which is considered the preferential site for nutrient exchange. Direct DNA extraction from the whole root and sequencing of ribosomal gene regions are commonly carried out to investigate intraradical AMF communities. Nevertheless, this protocol cannot discriminate between the AMFs that actively produce arbuscules and those that do not. To solve this issue, the authors have characterized the AMF community of arbusculated cells (AC) through a laser microdissection (LMD) approach, combined with sequencing-based taxa identification. The results were then compared with the AMF community that was found from whole root DNA extraction. The AMF communities originating from the LMD samples and the whole root samples differed remarkably. Five taxa were involved in the production of arbuscules, while two taxa were retrieved inside the root but not in the AC. Unexpectedly, one taxon was found in the AC, but its detection was not possible when extracting from the whole root. Thus, the LMD technique can be considered a powerful tool to obtain more precise knowledge on the symbiotically active intraradical AMF community.

## Introduction

Obligate symbiotic fungi that form arbuscular mycorrhizae (AMF; belonging to the phylum Glomeromycota) are among the most important soil microorganisms. AMFs facilitate mineral nutrient uptake from the soil in exchange for plant-assimilated carbon and promote water-stress tolerance and resistance to certain diseases (Smith and Read, 2008). About 240 AMF species have been described so far (Oehl et al., 2011; Krüger et al., 2012), primarily on the basis of spore morphology but increasingly supported by DNA sequence information. The use of next generation sequencing methods (e.g., pyrosequencing) has changed our understanding of the diversity of AMF, by allowing AM fungi to be directly detected from environmental samples (Öpik et al., 2009, 2013; Lumini et al., 2010; Dumbrell et al., 2011; Davison et al., 2012). Comparisons between AMF communities inside and outside the roots have shown that all the phylotypes from the roots were represented in the soil, which, as expected, maintains a higher AMF biodiversity than roots (Balestrini et al., 2010).

The AMF enters the root epidermis through the hyphopodium and starts to colonize the root by producing inter and intracellular hyphae. When the fungus penetrates the inner cortical cells, it produces a complex ramified structure called arbuscule, which is considered the preferential site for nutrient exchange (Smith and Read, 2008). It is rather easy, although quite time-demanding and subjective, to spot arbuscules inside root parenchyma cortex cells through staining and microscopy (Trouvelot et al., 1986). Unfortunately, it is rather difficult, if not impossible, to discriminate the specific taxa involved in the production of these structures according to the morphology, even at a high taxonomical rank level. In order to investigate intraradical AMF communities, direct DNA extraction from the whole root and sequencing of ribosomal gene regions are commonly carried out (Balestrini et al., 2010; Lumini et al., 2011; Borriello et al., 2012; Pellegrino et al., 2012). Nevertheless, this protocol is not able to discriminate between the AMFs that actively produce arbuscules and those that do not. Apart from arbuscules, a multitude of intraradical AMF structures are normally encountered in a host-root, i.e., hyphae, vesicles, spores, and hyphopodia, and they contain DNA but have no direct correlation with the plant-fungus exchange activity. Therefore, the amplification of DNA from such structures alters the actual shape of the active AMF community due to a dilution effect.

In an attempt to discriminate the active intraradical AMF community, the authors have proposed the characterization of the AMF community of arbusculated cells (AC) through a laser microdissection (LMD) approach combined with sequencing-based taxa identification. The idea of applying LMD to plant tissues was first advanced during the early years of the new millennium and its potential was reviewed by Day et al. (2005). As predicted by these authors, the LMD technique has resulted to be a powerful tool for isolating specific tissues and cell types from sectioned plant specimens, as it allows the cell specific extraction of RNA, DNA, and proteins/metabolites (Nelson et al., 2006; Balestrini and Bonfante, 2008). During the last few years, LMD has been used to study gene expression in AMF. Particular attention has been paid to specific plant and fungal genes expressed in the cortical cells containing arbuscules (Balestrini et al., 2007; Gomez et al., 2009; Guether et al., 2009; Gaude et al., 2011; Hogekamp et al., 2011; Pérez-Tienda et al., 2011; Tisserant et al., 2012). However, no information is available on the potential use of LMD for the identification of intraradical active AMF taxa on the basis of only DNA extraction from homogeneous cell type populations (AC). In the present work, the aim has been to isolate the specific fungal compartments that have a crucial symbiotic role in order to correctly identify functionally active AMF species.

In this study, *Camellia japonica* L. ‘Nuccio's Pearl’ was chosen as the experimental plant. This plant species is a broadleaf evergreen perennial shrub and it is difficult to consider it a model plant or agricultural crop, since very limited research information is available on the species. The camellia is an acidophilic plant and is characterized by a very specific rhizospheric chemistry because of the phenolic-rich root exudates and low pH characteristics that make this species a difficult plant material to work with. The rhizosphere of *C. japonica* and its close species has been demonstrated to host a diversity of microorganisms, including AMFs (Gupta and Sharma, 2010), bacteria (Pandey and Palni, 1996) and nematodes (Zheng et al., 2000). The authors have developed a method to extract DNA from microdissected arbuscule-containing cells from the mycorrhizal roots of potted camellias. After DNA amplification with AMF specific primers, the PCR products were cloned and sequenced. This led to the identification of the AM fungi that formed arbuscules inside the roots. These data were compared with the DNA sequences obtained from direct whole root DNA extraction in order to provide information on the AMF species that are involved in the formation of a functional symbiosis.

## Materials and methods

### Plant material and growth conditions

*C. japonica* ‘Nuccio's Pearl’ was selected as the test plant because of its commercial and ornamental value. The cultivation cycle lasted almost 2 years (February 2011–November 2012) and was carried out in a commercial nursery devoted to the production of acidophilic ornamental plants (Tecnoverde s.p.a., Verbania, Piedmont—Italy). A substrate mixture with 50% of coconut fiber and 50% of standard substrate (89% commercial *Sphagnum* peat and 11% agriperlite) was used as the growing medium for all the plants. This substrate had a pH of 5.6 (substrate/water ratio: 1/5 v/v), an electrical conductivity (EC) of 38 μS/cm and a C/N ratio of 76.7. The K, Ca, and Mg contents were 0.1, 0.4 and 0.1%, respectively, while no detectable concentrations of P were present. Neither a spore count nor a PCR-based approach with specific primers (see the Materials and Methods section below) showed the presence of AMF propagules in the substrate mixture. A fertilizer dosage of 2.25 kg m^−3^ CaCO_3_, 1.12 kg m^−3^ Osmocote Exact® (15N-3.9P-7.5K + trace micronutrients, 8–9 months, Scotts, Merysville, Ohio) and 0.5 kg m^−3^ Scorie Thomas (6.9P, phosphatic inorganic fertilizer, Timac Italia, Ripalta Arpina, Italy) were added to the substrate mixture. In the first week of February 2011, three camellia rooted cuttings were planted per pot (9 cm in diameter) for a total of 35 pots (105 rooted cuttings), and 100 g of AMF inoculum was added to each pot (see below for the methods regarding the inoculum formulation). The pots were randomly distributed over a bench in a frost-free greenhouse (2.5°C minimum, 16°C average). The shading was set to a constant 30%. 40% additional shading was provided when the natural illumination exceeded 60 Klux. No supplementary lighting was applied. All the camellias were watered and fertigated with 20N-8.7P-16.6K Peter Professional hydrosoluble fertilizer (Scotts, Merysville, Ohio) at 0.8–0.9 g l^−1^ about once every 2 weeks from March to October, according to the weather conditions. After 9 months of cultivation, the plants were transplanted into 15 cm diameter pots and pruned. The cultivation ended in November 2012, when the plant roots were harvested and stored at −80°C or immediately processed.

### Inoculum preparation

The bulk soil surrounding centennial camellia specimens in the public garden on the Isola Madre isle (45° 54′ 43.46″ N, 8° 32′ 21.35″ E, Verbania, Italy) was collected on May 22nd 2009. A physicochemical analysis was performed in three replicates to characterize the soil properties, according to European Standard methods (EN). The soil collected had a pH of 5.9, an EC of 62.7 μ S/cm, and very low porosity (9%). The C/N ratio was 13.1, the total P was 0.02% while the K, Ca, and Mg were 0.45, 0.16, and 4.86%, respectively. The sampling operations consisted in digging to the first 5–20 cm and collecting bulk soil (ca 1.5 Kg) and fine feeder roots belonging to a centennial *C. japonica* ‘Alba Plena’ tree. The bulk soil and roots were taken to the lab, gently homogenized and mixed with 60% sterile sand. The resulting substrate was used as growing media for trap plants in order to propagate the AMF portion present in the inoculum. The species used for this purpose were *Nicotiana tabacum, Lolium perenne, Trifolium repens*, and *Plantago major*, all of which are recognized as being highly efficient trap plants (Liu and Wang, 2003; Oehl et al., 2004). After 9 months, the AMF inoculum was re-propagated once again by homogenizing the trap plant substrate and mixing it with 60% sterile sand. The amount of inoculum required to carry out the experiment was obtained after another 9 months. The total inoculum, including chopped root systems, was homogenized and air-dried at room temperature. A portion of roots belonging to the trap plant species was washed free of the substrate, air-dried at room temperature and immediately used for a morphological assessment of mycorrhization levels, according to the protocol described by Trouvelot et al. (1986). The mycotrophic status of the root samples was consistent, the frequency of mycorrhization was more than 85%, the intensity of colonization was around 30%, and the presence of arbuscules in the mycorrhizal roots amounted to 70%. In order to evaluate the infectiveness of the inoculum, the Most Probable Number (MPN, Porter, 1979) of AMF propagules was calculated at 130 propagules/cm^3^.

### Molecular characterization of the AMF inoculum

Before starting the experimentation on the camellia plants, a molecular AMF community composition study was performed on the crude AMF inoculum, in order to characterize all the isolates that had propagated successfully. DNA extraction was performed on the trap plant roots and on the crude inoculum, as reported by Borriello et al. (2012), according to the protocols for frozen root samples and bulk soil, respectively. A nested PCR approach (Borriello et al., 2012) was adopted to amplify a region of the small subunit (SSU) of the Glomeromycota ribosomal DNA. This approach consisted of a first amplification with the universal eukaryotic primers NS1 and NS4 (White et al., 1990) and a following amplification round on the 100X diluted PCR product with Glomeromycota-specific primers AML1 and AML2 (Lee et al., 2008). PCR was carried out using 0.2 mM dNTPs, 3.5 mM of MgCl_2_, 0.5 μM of each primer and the supplied reaction buffer, with two units of High Fidelity Taq (ROCHE) to obtain a final volume of 20 μl. Amplifications were carried out in 0.2 ml PCR tubes using a Biometra T Gradient thermocycler, according to the following steps: 5 min initial denaturation at 95°C; 35 cycles of 1 min at 95°C, 1 min at 55°C and 58°C for the two nested PCR rounds, respectively, 1 min at 72°C; and a final elongation of 10 min at 72°C. A negative control was included in the PCR to check for contamination. All the PCR products were checked using 1.2% agarose gel stained with Ethidium Bromide (Sigma-Aldrich). The PCR products were purified using QIAquick (Qiagen, Hilden, Germany), cloned in a pGEM-T Easy vector (Promega, Madison, Wisconsin, USA) and transformed into competent *Escherichia coli* cells (Xl1 blue). After colony PCR, inserts of approximately the correct expected size (~800 bp) were submitted to restriction fragment length polymorphism (RFLP) analysis using two endonucleases, Hsp92II (|CATG|) and HinfI (G|ANT|C), in order to reduce the number of clones to be sequenced. Subsequently, 1–2 clones were selected as being representative of each found RFLP profile and were sequenced using T7 vector primers by Macrogen sequencing services (Macrogen, Seoul, Korea). Sequence editing was conducted using SEQUENCER V4.2.2 (Gene Codes Corporation, Ann Arbor, MI, USA). The potential chimera sequences were identified using the Chimera Detection programme (Cole et al., 2003). A search for similar sequences was conducted with the BLAST tool (Zhang et al., 2000), provided by GenBank. All the sequences were aligned using the multiple sequence comparison alignment tool by MAFFT version 6 (Katoh and Toh, 2008) and a Neighbor-joining (NJ) phylogenetic analysis (5000 bootstrap replicates) was performed with MEGA version 5 (Tamura et al., 2011). Sequences of the Paraglomerales AMF order were set as the outgroup. A Maximum Likelihood phylogenetic analysis (1000 bootstrap replicates) was also conducted and comparable results were obtained.

### Assessment of the inoculated root colonization by AM fungi

A portion of inoculated camellia roots from three randomly selected potted plants was washed free of the substrate, air-dried at room temperature and chopped into small fragments that were cleared twice with 10% KOH for 30 min at 90°C. After rinsing several times with tap water, the root fragments were transferred to alkaline water (H_2_O:NH_4_OH:H_2_O_2_ 198:1:1) for 60 min, stained with 0.1% cotton blue in 80% lactic acid for about 18 h and then de-stained several times with 80% lactic acid washes. The roots were cut into 1 cm fragments and placed onto microscope slides. 25 fragments were observed for each replicate, for a total of 75 root fragments. Mycorrhiza frequency (F%), root AMF colonization intensity in the mycorrhizal root parts (m%) and in the whole root system (M%), and the presence of arbuscules in mycorrhizal root parts (a%) and in the whole root system (A%) were determined and calculated, as described by Trouvelot et al. (1986).

### DNA extraction from the whole root and laser microdissected cells

**Figure 1:**
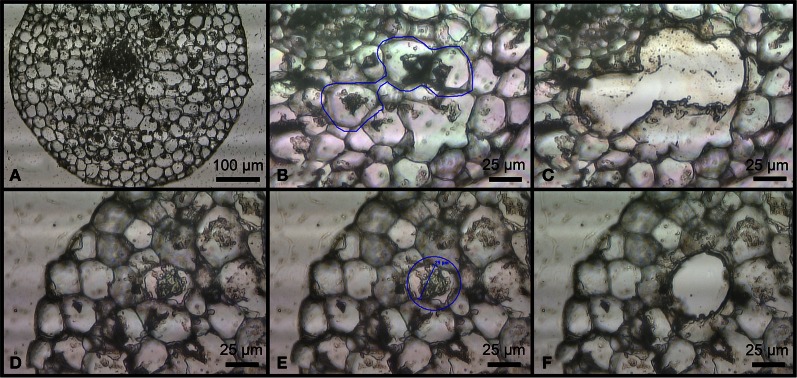
**Sections of different camellia roots on a Leica RNase-free PEN-foil slide after paraffin embedding.** Arbuscule-like structures located on a transversal root section **(A)**. Transversal root section before **(B)** and after **(C)** multiple contiguous arbuscules were collected using LMD. Transversal root section before **(D,E)** and after **(F)** a single arbuscule was collected using LMD.

DNA extraction on the whole root was performed in duplicate (abbreviated as sample R1 and R2) with the DNeasy Plant Mini Kit (Qiagen, Crawley, UK), on 0.1 g of finely ground frozen root samples taken from three randomly chosen camellia pots. In order to prepare the plant material for microdissection, the roots from the same camellia pots were fixed in freshly prepared absolute ethanol:acetic acid (3:1) at 4°C overnight for paraffin embedding (Balestrini et al., 2007). A Leica AS LMD system (Leica Microsystems) was used to isolate the arbuscule-containing cells from the prepared tissue sections, as described in previous works (Balestrini et al., 2007; Guether et al., 2009). Three different aliquots containing 100, 300, and 500 putative AC (abbreviated as A100, A300, and A500, rispectively) were collected in 0.2 ml eppendorf tube caps from slides filled with root transversal and longitudinal 11–12 μm root sections obtained from at least 10 different root fragments (Figure 1). After collection, the samples were briefly centrifuged to move the plant material at the bottom of the tubes and a DNA extraction buffer (including Proteinase K) from the Arcturus PicoPure DNA Extraction kit (Life Technologies) was added. The samples were gently vortexed and incubated at 65°C for 3 h. In order to inactivate Proteinase K, the samples were placed at 95°C for 10 min. After DNA extraction, yield was measured using a NanoDrop 1000 spectrophotometer.

### Nested PCR, cloning, RFLP, and sequencing of the fungal ribosomal (rRNA) gene

Nested PCR, cloning, and RFLP typing were carried out for whole root or microdissected arbusculated cell samples with the same approach adopted for the inoculum molecular characterization. Two microliter of the undiluted DNA was used as input during the first round of nested PCR for the microdissected arbusculated cell samples and 2 μl of the 1:20 diluted DNA was used for the whole root samples. The only modification consisted of the use of an undiluted PCR product as a template for the second round of PCR when the microdissected samples were considered. The same way as during the characterization of the inoculum, after colony PCR, inserts of approximately the correct size from each SSU DNA library were RFLP typed by comparing the obtained bandage with the RFLP profile data found during the previous molecular characterization of the inoculum. 24 representative samples were sequenced, using primer pair AML1-AML2, by Sequencing Service at Munich University (Database ID: http://www.gi.bio.lmu.de/sequencing). The sequences were edited and their affiliation investigated, as described in the above sections. In order to assess the reliability of the community analysis, the representativeness of the observed community distribution was calculated, with respect to the real community distribution in the sample, with the Estimated Sample Coverage (ESC) and the bias corrected CHAO1 species richness estimator, by using the freeware SPADE (Chao and Shen, 2010). In addition, rarefaction curves were built with PAST version 2.17 (Hammer et al., 2001).

## Results

### AMF inoculum composition

**Figure 2:**
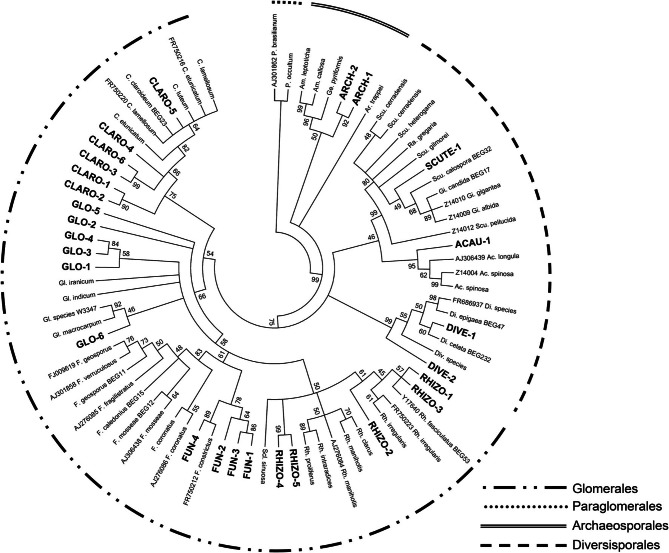
**The phylogenetic tree constructed on a portion of the arbuscular mycorrhizal fungal SSU rDNA (~800 bp) using the Neighbour-Joining method (Saitou and Nei, 1987).** The percentage of replicate trees in which the associated taxa clustered together in the bootstrap test (5000 replicates) is shown next to the branches (Felsenstein, 1985). Branches corresponding to partitions reproduced in less than 45% bootstrap replicates were collapsed. The evolutionary distances were computed using the Kimura 2-parameter method (Kimura, 1980) and are in the units of the number of base substitutions per site. The analysis involved 79 nucleotide sequences (52 reference sequences and 27 sequences representative of the found RFLP profiles, highlighted in bold black). Evolutionary analyses were conducted in MEGA5 (Tamura et al., 2011). The external brackets indicate diverse taxonomic groups, as described by Krüger et al. (2012). The Paraglomerales order was used as the outgroup.

Fifty-three transformed clones were sequenced as representatives of each of the 35 RFLP profiles that were found. The phylogenetic inference (Figure 2) revealed that, of the 35 obtained RFLP profiles, 27 (45 sequences) clustered with major taxonomical groups belonging to the Glomeromycota phylum, i.e., Archaeosporales, Glomerales, and Diversisporales. The remaining 8 RFLP profiles (8 sequences) were mainly related to sequences from plant and other soil organisms, as they were the result of aspecific amplifications.

### Mycotrophic status of inoculated camellia roots

**Figure 3:**
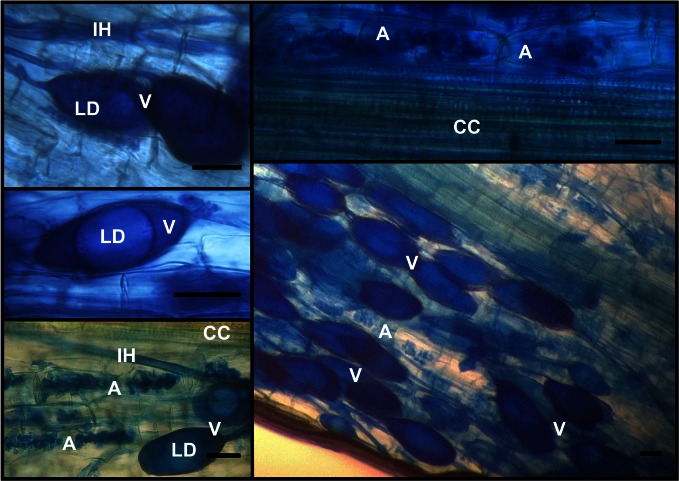
**Typical AMF structures, stained with 0.1% cotton blue, encountered in *C. japonica* ‘Nuccio's Pearl’ roots.** Intraradical hyphae (IH), along with intraradical vesicles (V) that were recognized because of the presence of lipid droplets (LD), pervaded the root apparatuses. Intracellular arbuscules (A) colonized large patches of the root cortex, as expected, without entering the central cylinder (CC). The bars stand for 30 μm.

Most of the camellia root fragments exhibited a very high degree of AMF colonization. The mean percentage value of the frequency was 90.0 ± 7.3, while the percentage intensity of the colonization calculated in the root system and in the root fragments was 57.1 ± 6.2 and 63.1 ± 1.7, respectively. Arbuscules covered 54.9 ± 5.6% of the total root system. In particular, 96.4 ± 2.2% of the mycorrhizal parts hosted these structures. Typical symbiotic scenarios are portrayed in Figure 3.

### AMF detection in the arbusculated cells and whole root samples

**Figure 4:**
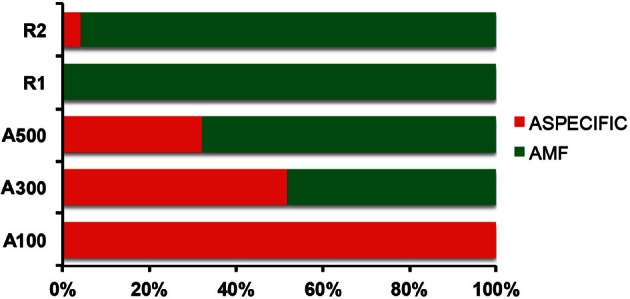
**Percentage of aspecific amplification detected in the whole root samples (R1 and R2) and from 100 (A100), 300 (A300), and 500 (A500) arbusculated cell aliquots, respectively**.

**Figure 5:**
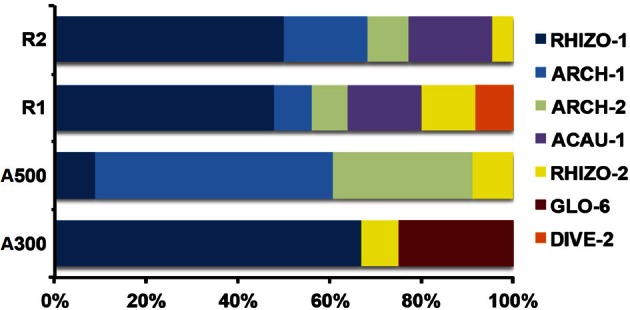
**RFLP profile percentage distribution for the AMF communities in the whole root samples (R1 and R2) and in the 300 (A300) and 500 (A500) arbusculated cell aliquots**.

**Figure 6:**
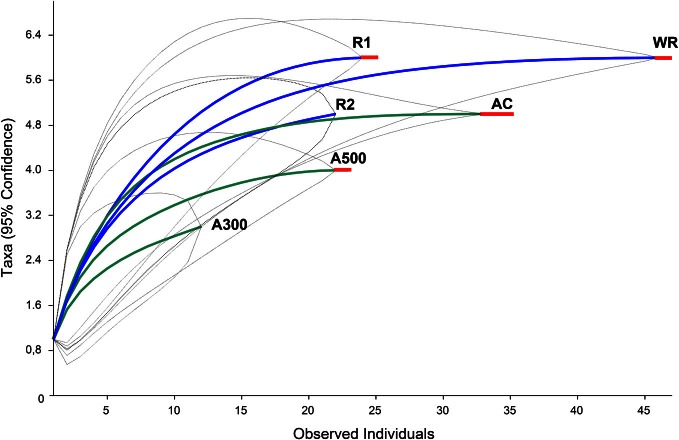
**Rarefaction curves indicating RFLP profile accumulation related to the number of screened clones for the LMD samples (A300, A500), whole root samples (R1, and R2) and respective cumulative samples (AC and WR).** The dotted lines indicate the upper and lower 95%-confidence intervals. The red lines indicate that the curve reached the asymptote.

**Figure 7:**
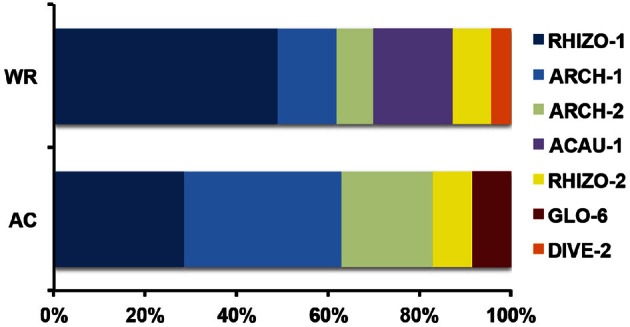
**RFLP profile percentage distribution for the whole root cumulative community (R1 + R2 = WR) and the arbusculated cell cumulative community (A300 + A500 = AR)**.

The DNA extractions yielded 25.8, 29.2, and 27.9 ng μl^−1^ for the LMD samples (A100, A300, A500) and 20.5, and 26.0 ng μl^−1^ for the whole root samples (R1 and R2), respectively. The nested PCR, using the AML1-AML2 primer combination, yielded a clone library that contained SSU fragments from the whole root and arbusculated cell matrices for each sample under study. 120 clones were screened and RFLP typed. The presence of 13 different RFLP profiles was highlighted and 24 representative samples (1–3 per RFLP profile) were sequenced. Apart from sequences showing high similarity (>97% identity) with AM fungi (Glomeromycota phylum), sequences corresponding to non-target organisms were also obtained, as already shown during the inoculum molecular characterization. BLAST searches revealed that, of the 24 obtained SSU sequences, 16 showed high similarity to sequences from taxa belonging to the Glomeromycota phylum, while the remaining eight were mainly related to sequences from other organisms (e.g., *C. japonica* and other fungi) and were the result of an aspecific amplification of primer pair AML1-AML2. The percentage of screened clones containing a non-AMF SSU DNA fragment is shown in Figure 4. None of the clones screened from the SSU DNA library belonging to the aliquot containing 100 arbusculated cells (A100) contained an AMF DNA fragment. The DNA extracted from the aliquots containing a higher number of cells, 300 (A300) and 500 (A500), showed a proportional increase in terms of AMF SSU fragments (around 50 and 70%, respectively). The SSU DNA libraries from the whole root samples (R1 and R2) showed little or no presence of non-AMF fragments. After discarding all the non-target fragments, clones showing the same RFLP profiles were affiliated to an AMF phylogenetic group on the basis of BLAST search results, following the same procedure adopted during the inoculum molecular characterization. As a result, an AMF community distribution was obtained for each sample (Figure 5). The AMF community of sample A300 was characterized by 12 observed individuals and the presence of 3 RFLP profiles, RHIZO-1, the most abundant (66.7%), GLO-6 (25%), and RHIZO-2 (8.3%). The ESC for this sample was 91.7% but the rarefaction curve did not reach the asymptote (Figure 6), while bias corrected CHAO1 (3.0 ± 0.4) confirmed the reliability of the number of found RFLP profiles. In A500, the community counted 23 observed individuals distributed over 4 RFLP profiles, ARCH-1, the most abundant (52.2%), ARCH-2 (30.4%), RHIZO-1 (8.7%), and RHIZO-2 (8.7%). The sample coverage was maximum (ESC = 100%), the related rarefaction curve reached the asymptote (Figure 6) and bias corrected CHAO1 (4.0 ± 0.0) confirmed the representativeness of the observed community. Only 2 RFLP profiles (RHIZO-1 and RHIZO-2) were found in both samples. The whole root communities were more uniform with as many as five shared RFLP profiles. In R1 (25 observed individuals) and R2 (22 observed individuals), RHIZO-1 was the most abundant RFLP profile (48 and 50%, respectively). Another 4 RFLP profiles were shared, ACAU-1 (16 and 18.2%), ARCH-1 (8 and 18.2%), ARCH-2 (8 and 9.1%), and RHIZO-2 (12 and 4.5%). One RFLP profile, DIVE-2, was only found in R1 (8%). ESC was 100% for R1 and 95.5% for R2. Bias corrected CHAO1 gave 6.0 ± 0.0 and 5.0 ± 0.3 expected RFLP profiles for R1 and R2, respectively. Only for sample R2, was the asymptote not reached by the related rarefaction curve (Figure 6). Overall, the cumulative AMF communities from the whole root (WR) and LMD AC shared 4 RFLP profiles, RHIZO-1, ARCH-1, ARCH-2, and RHIZO-2 (Figure 7). In this case, the rarefaction curves related to both cumulative communities reached the asymptote (Figure 6).

## Discussion

To the authors' knowledge, this experiment has been the first attempt to characterize an arbuscule forming AMF community through the use of LMD. Previous studies on AM symbiosis using LMD were aimed at investigating gene expression on AMF isolates that were inoculated singularly in microcosm conditions (Balestrini et al., 2007; Guether et al., 2009; Giovannetti et al., 2012). In the present study, a mesocosm approach has been adopted. The used inoculum originated from natural soils and it is therefore likely that the AMF species that were present were propagated along with other rhizosphere biota.

Prior to DNA extraction, three aliquots, containing 100, 300, and 500 AC (A100, A300, and A500, respectively), were prepared by dissecting them from root sections from at least 10 different root fragments. The nested PCR approach, using the primer pair AML1-AML2, gave a positive band of averagely 800 bp for all three samples, as expected. After cloning and RFLP typing, a sequencing step made it possible to characterize the taxonomical origin of the DNA amplicons. All the sequences retrieved from sample A100 were the result of aspecific amplifications. This was possibly due to the fact that this primer pair (Lee et al., 2008) as well as other primer pairs in general, although said to be Glomeromycota-specific, can sometimes lead to aspecific amplification when the starting template resides in a complex matrix and is overwhelmed by non-target DNA (Douhan et al., 2005; Alguacil et al., 2008; Toljander et al., 2008; Borriello et al., 2012). Samples A300 and A500 gave decreasing rates of non-target amplification, while aspecific amplification was absent or negligible for the whole root samples (R1 and R2). Around 50% of the screened clones in sample A300 were shown to be carrying a non-target fragment and this led to a decreased number of total observed AMF individuals (13). This factor was clearly responsible for the lower sample coverage achieved for this sample (ESC = 91.7%, with the rarefaction curve not reaching the asymptote, Figure 6). The reciprocal discrepancies observed in terms of RFLP profile presence/abundance (RHIZO-1, ARCH-1, ARCH-2, and GLO-6) between A300 and A500 suggest that these two samples singly cannot be regarded as 100% representative of the actual arbuscule forming community. This could be due to a lack of robustness in the sampling effort, although the coverage and species richness estimators denoted a remarkable representativeness of the sample communities. It is possible that the complexity of the mesocosm conditions may have triggered the development of a heterogeneous rhizosphere biota. This could have played a critical role in diminishing or making AMF detection impossible in smaller cell aliquots. The spectrophotometer measurements of the DNA extraction yields from the different cell aliquots showed comparable values and did not differ remarkably from the ones performed on the whole root extractions. However, these values refer to total DNA and may not reflect the actual presence of target AMF DNA in the sample. Moreover, AC were collected on a morphological basis and, to avoid an impact on DNA preservation, no staining was performed to aid their discrimination from other non-AMF structures. Thus, further studies will require a greater effort in terms of number of AC collected from roots sampled from mesocosm or natural conditions. As an alternative, new and more specific primer pairs could be designed and their use consolidated, in order not to lose AMF-specificity due to the overwhelming pressure exerted by non-target organisms that are present in complex matrices. The use of DNA conservative fungal staining techniques (Pitet et al., 2009) should also be considered, in order to assist arbusculated cell individuation.

In general, the interaction between the involved symbiotic partners was significant as nearly every mycorrhizal root part checked presented a profuse arbuscule production. This, in combination with a high frequency and intensity of colonization, made it easy to collect suitable root fragments for LMD use, as the technique relies mainly on the degree of availability and morphological detectability of intracellular arbuscules. Overall, out of the 27 taxa inoculated in the substrate (see Materials and Methods), only 5 were recovered from microdissected AC. This is an important result, as it could suggest that the related 5 taxa are capable of undertaking a close partnership with the plant species under study and which actively express their functional traits in terms of benefits for plant fitness. A large number of intraradical vesicles were also found all along the root system (Figure 3). Vesicles can carry a comparable number of nuclei to that of the spores (Gamper et al., 2008) and can therefore be detected through PCR with specific primers. This may lead to an overdetection of the fungal isolates that produce abundant intraradical vesicles, when the AMF community composition of the whole root is being investigated. This could be the case of the fungal isolate affiliated to RFLP profile RHIZO-2, which clustered, according to the phylogenetic tree, with a sequence belonging to *Rhizophagus fasciculatum* (Figure 2) and was the most abundant type found in the roots (Figure 7). This AMF species is characterized by profuse vesicle formation (Gerdemann and Trappe, 1974) and the greater presence of gDNA belonging to this taxon in the root samples might therefore have hindered or precluded the molecular recovery of other coexisting taxa. This is the case of ARCH-1 and ARCH-2, which were less abundant in the whole root community with respect to the community that featured AC. These RFLP profiles grouped with sequences affiliated to the Archaeosporales order, a taxonomical rank that includes species that form rare or no vesicles (Morton and Redecker, 2001; Walker et al., 2007). The same considerations can be made for GLO-6, which was not found in the whole root samples but was, albeit scarcely, detectable in the community from the LMD AC. Possible reasons for this could be that the small patches infected by this fungus along the roots were not spotted after whole root DNA extraction, due to the overwhelming presence of DNA of other more abundant taxa, but were instead detected through LMD. The explanation could be that this technique, being based only on the collection of intracellular arbuscules, can eliminate the co-extraction of DNA from other fungal structures (vesicles, spores, hyphae) that exert a dilution effect on the actual arbuscule forming community. Therefore, it is likely that, thanks to LMD, candidate beneficial AMFs that colonize small patches within the root, but which play a pivotal role in exploring the surrounding soil (Parniske, 2008), can be spotted. Interestingly, two RFLP profiles, i.e., ACAU-1 and DIVE-2, were highlighted in the whole root samples but not in the LMD samples, indicating that they could be located within the root but might not produce intracellular arbuscules. This finding would seem to support the hypothesis that the production of arbuscules by different AMF taxa could be differentially stimulated according to their momentary state of efficiency. Conceptually, it is feasible that ACAU-1 and DIVE-2 could be AMF species that were momentarily expressing less beneficial attributes for the plant at the moment of sampling. Interestingly, recent findings have shown how AM symbiosis, depending on the partners involved, can range from cooperative to less-cooperative (Verbruggen and Kiers, 2010; Kiers et al., 2011). However, to provide supporting experimental evidence, further analyses are necessary to investigate the functional traits involved in AM symbiosis.

In conclusion, the LMD technique can be considered a powerful tool for the obtainment of a more qualitative representation of the arbuscule forming and, therefore, symbiotically active AMF community. Future studies on LMD-mediated identification of active AMF should focus on the field validation of this method for AMF traceability studies and could be finalized on the formulation of selected inocula for field and greenhouse applications. Further efforts should be devoted to LMD-mediated functional biodiversity studies, in which community composition analyses based on functional genes, e.g., the phosphate transporter gene, will be able to shed light on the beneficial traits involved in AM symbiosis.

### Conflict of interest statement

The authors declare that the research was conducted in the absence of any commercial or financial relationships that could be construed as a potential conflict of interest.
